# Anterior mitral isthmus line using pulsed-field ablation with the pentaspline catheter or radiofrequency ablation: procedural characteristics, safety, and mid-term outcomes

**DOI:** 10.1093/europace/euaf265

**Published:** 2025-10-23

**Authors:** Jonas Brügger, Corinne Isenegger, Fabian Jordan, Behnam Subin, Reto Stump, Sven Knecht, David Spreen, Nicolas Schaerli, Philipp Krisai, Beat Schaer, Felix Mahfoud, Christian Sticherling, Michael Kühne, Patrick Badertscher

**Affiliations:** Department of Cardiology, University Hospital Basel, Petersgraben 4, Basel 4031, Switzerland; Cardiovascular Research Institute Basel, University Hospital Basel, Petersgraben 4, Basel 4031, Switzerland; Department of Cardiology, University Hospital Basel, Petersgraben 4, Basel 4031, Switzerland; Cardiovascular Research Institute Basel, University Hospital Basel, Petersgraben 4, Basel 4031, Switzerland; Department of Cardiology, University Hospital Basel, Petersgraben 4, Basel 4031, Switzerland; Cardiovascular Research Institute Basel, University Hospital Basel, Petersgraben 4, Basel 4031, Switzerland; Department of Cardiology, University Hospital Basel, Petersgraben 4, Basel 4031, Switzerland; Cardiovascular Research Institute Basel, University Hospital Basel, Petersgraben 4, Basel 4031, Switzerland; Department of Cardiology, University Hospital Basel, Petersgraben 4, Basel 4031, Switzerland; Cardiovascular Research Institute Basel, University Hospital Basel, Petersgraben 4, Basel 4031, Switzerland; Department of Cardiology, University Hospital Basel, Petersgraben 4, Basel 4031, Switzerland; Cardiovascular Research Institute Basel, University Hospital Basel, Petersgraben 4, Basel 4031, Switzerland; Department of Cardiology, University Hospital Basel, Petersgraben 4, Basel 4031, Switzerland; Cardiovascular Research Institute Basel, University Hospital Basel, Petersgraben 4, Basel 4031, Switzerland; Department of Cardiology, University Hospital Basel, Petersgraben 4, Basel 4031, Switzerland; Cardiovascular Research Institute Basel, University Hospital Basel, Petersgraben 4, Basel 4031, Switzerland; Department of Cardiology, University Hospital Basel, Petersgraben 4, Basel 4031, Switzerland; Cardiovascular Research Institute Basel, University Hospital Basel, Petersgraben 4, Basel 4031, Switzerland; Department of Cardiology, University Hospital Basel, Petersgraben 4, Basel 4031, Switzerland; Cardiovascular Research Institute Basel, University Hospital Basel, Petersgraben 4, Basel 4031, Switzerland; Department of Cardiology, University Hospital Basel, Petersgraben 4, Basel 4031, Switzerland; Cardiovascular Research Institute Basel, University Hospital Basel, Petersgraben 4, Basel 4031, Switzerland; Department of Cardiology, University Hospital Basel, Petersgraben 4, Basel 4031, Switzerland; Cardiovascular Research Institute Basel, University Hospital Basel, Petersgraben 4, Basel 4031, Switzerland; Department of Cardiology, University Hospital Basel, Petersgraben 4, Basel 4031, Switzerland; Cardiovascular Research Institute Basel, University Hospital Basel, Petersgraben 4, Basel 4031, Switzerland; Department of Cardiology, University Hospital Basel, Petersgraben 4, Basel 4031, Switzerland; Cardiovascular Research Institute Basel, University Hospital Basel, Petersgraben 4, Basel 4031, Switzerland

**Keywords:** Atrial fibrillation, Pulmonary vein isolation, Anterior line, Mitral isthmus line, Pulsed-field ablation, Radiofrequency ablation

## Abstract

**Aims:**

Pulsed-field ablation (PFA) is a non-thermal energy source for pulmonary vein isolation (PVI), offering advantages in safety and procedural efficiency. However, data comparing anterior mitral isthmus line (MIL) ablation using PFA vs. conventional radiofrequency ablation (RFA) are scarce. This study aimed to compare procedural characteristics, safety, and arrhythmia recurrence following PVI with additional anterior MIL ablation using PFA vs. RFA.

**Methods and results:**

In this prospective, single-centre analysis from the SWISS-AF-PVI registry, 129 patients (median age 70 years, 40% female) undergoing PVI with anterior MIL ablation were included. Patients received either PFA with a pentaspline catheter (*n* = 61) or RFA using a 3.5 mm irrigated tip catheter (*n* = 68). Procedural parameters, complications, and arrhythmia recurrence were assessed over a median follow-up of 327 days. PFA significantly reduced total procedure time (71 vs. 108 min, *P* < 0.001), LA dwell time (53 vs. 80 min, *P* < 0.001), and ablation time (27 vs. 50 min, *P* < 0.001) compared to RFA. MIL ablation with PFA required fewer applications (14; 35 s vs. 473 s RFA, *P* < 0.001). Arrhythmia-free survival was similar between groups (PFA 48.8% vs. RFA 61.8%, *P* = 0.34). Among 34 patients undergoing redo procedures, incomplete MIL was found in 53%, with no significant difference between groups. Three major complications occurred.

**Conclusion:**

Anterior MIL ablation using PFA is feasible, safe, and more time-efficient than RFA, with comparable mid-term arrhythmia outcomes. However, high rates of MIL reconnection and arrhythmia recurrence highlights the need to improve lesion durability.

## Graphical Abstract

**Graphical Abstract:**
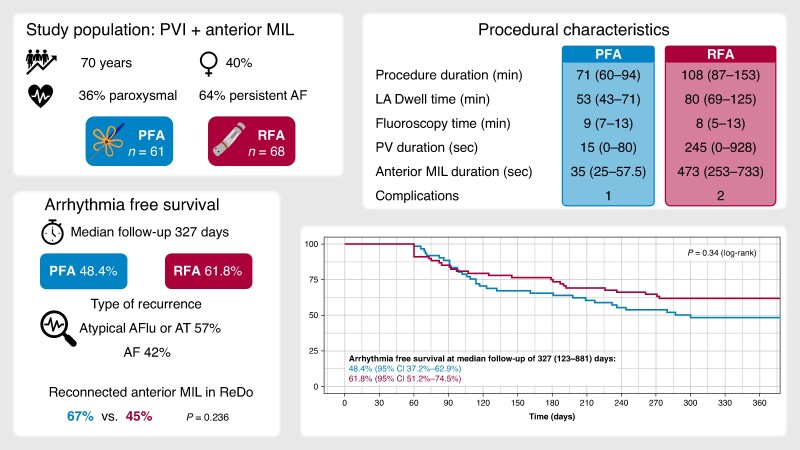
AFlu, atrial flutter; AF, atrial fibrillation; AT, left atrial tachycardia; LA, left atrial; MIL, mitral isthmus line; PFA, pulsed-field ablation; PV, pulmonary vein; RFA, radiofrequency ablation.

What’s new?

- This study prospectively compared pulsed-field ablation (PFA) vs. radiofrequency ablation (RFA) for pulmonary vein isolation with additional application of an anterior mitral isthmus line (MIL).
- PFA substantially reduced procedure duration, left atrial dwell time, and ablation time compared with RFA, confirming its efficiency advantage also for linear lesions.
- Despite procedural efficiency, arrhythmia-free survival and MIL reconnection rates were comparable between PFA and RFA.
- These findings highlight the current limitations of linear lesion durability, irrespective of energy source, and emphasize the need for improved catheter designs.

## Introduction

Pulmonary vein isolation (PVI) is a cornerstone in the treatment of atrial fibrillation (AF) and has demonstrated superior efficacy over antiarrhythmic drug therapy, particularly in patients with paroxysmal AF.^1–3^ However, AF recurrence remains common, occurring in up to 40% of patients following ablation.^4^

The management of AF recurrences, especially in the setting of repeat catheter ablation, remains less well defined. Although pulmonary vein (PV) reconnection is widely recognized as a major contributor to AF recurrence, additional arrhythmogenic substrates beyond the PVs have been increasingly identified. The most common targets nowadays include the posterior wall and the mitral isthmus (MI).^5–9^

The emergence of pulsed-field ablation (PFA), a non-thermal ablation modality, has led to widespread adoption due to its favourable safety profile, rapid lesion delivery, and tissue selectivity.^10^ Its ease of use has encouraged operators to perform extensive substrate modification during both index and redo ablation procedures—often in the absence of high-level evidence. While posterior wall isolation (PWI) using PFA has been reported with promising acute success,^11,12^ data on PFA for linear ablation, particularly for mitral isthmus line (MIL) creation, are sparse.^13,14^ Furthermore, direct comparisons between MIL ablation with radiofrequency ablation (RFA) and PFA are lacking, especially regarding procedural efficiency, lesion durability, and clinical outcomes.

This study aims to compare procedural characteristics, safety, and mid-term outcomes in patients undergoing PVI with additional anterior MIL ablation using either PFA or RFA.

## Methods

### Patient population

This analysis included prospectively enrolled patients from the SWISS-AF-PVI registry (NCT03718364) with paroxysmal or persistent AF undergoing a first or repeat catheter ablation with PVI and additional application of an anterior MIL at a Swiss tertiary centre. Exclusion criteria were patients under 18 years, ablation within the coronary sinus for MIL or vein of Marshall (VOM) ethanol ablation. The decision to perform an anterior MIL was (i) the presence of MI-dependent atrial flutter at the beginning of the procedure, (ii) patients undergoing repeat catheter ablation with durable isolated PV, or (iii) the presence of atrial scar at the anterior wall of the left atrium (LA). Patients were informed about the risks and benefits of catheter ablation, including the possibility of additional lesion sets if deemed clinically appropriate. All patients provided written informed consent preprocedural. The study was approved by the local ethics committee and adhered to the declaration of Helsinki.

### Procedure

#### Preprocedural care

All patients underwent preprocedural transoesophageal echocardiography to rule out intracardiac thrombus and preprocedural computed tomography or magnetic resonance imaging for LA imaging. All procedures were performed under uninterrupted oral anticoagulation.

#### Pulmonary vein isolation

PVI was performed under sedation using midazolam, fentanyl and propofol by five experienced electrophysiologists. Ultrasound guidance was used for the femoral access and for the transseptal puncture (TSP) fluoroscopic guidance or in selected cases intracardiac or transoesophageal echocardiographic guidance. During the intervention an activated clotting time of 350 s was maintained by administering intravenous heparin.

#### Pulsed-field ablation

PFA was performed as previously described.^15^ In brief, the FARAPULSE PFA system (FARAPULSE ™, Boston Scientific, Natick, MA, USA) consists of (i) a 12F over-the-wire multipolar ablation catheter (FARAWAVE ™) available in sizes of 31 and 35 mm; (ii) a 13-F inner diameter steerable sheath (FARADRIVE ™); and (iii) a generator (FARASTAR ™).

In PFA cases, a parasympatholytic agent was administered intravenously post-TSP to prevent vagal response, and a 0.035-inch J-tip guidewire was used to cannulate the left superior pulmonary vein (LSPV) for PFA catheter deployment into the LA. PVI was performed with four PFA applications in a basket configuration and four in a flower configuration per vein, each delivering a maximum of 2 kV. After the first two applications in each configuration, the catheter was rotated by 30°–40° between pairs of applications to ensure full circumferential coverage of the vein. Two additional lesions for the right-sided carina between the superior and inferior vein were applied. The procedural endpoint of the PFA procedure was acute PVI success, assessed either by (i) 3D electroanatomic mapping (EAM) system (CARTO3, Biosense Webster, Irvine, CA, USA) of the LA with a multipolar mapping catheter (Octaray) or (ii) by entrance and, if deemed necessary (e.g. due to residual far-field atrial signal), exit block testing through the PFA catheter by pacing with 10 V and a pulse width of 2.0 ms for all electrodes. In repeat catheter ablation cases only reconnected PV (confirmed by EAM) were reisolated, until bidirectional block was achieved.

#### Anterior MIL with PFA

Anterior MIL ablation was performed using a series of lesions in a ‘flower’ pattern, extending from the mitral annulus to the right superior pulmonary vein (RSPV). The procedure was anatomically guided by fluoroscopy in the left anterior oblique view, along with a 3D-EAM system. Lesions were delivered until bidirectional block across the MI was achieved. MI block was confirmed by 3D-EAM. Preventive nitroglycerin administration was not systematically used in this cohort.

#### Radiofrequency ablation

RFA was performed as previously described.^16^ In brief, a multipolar mapping catheter (Octaray) or only the ablation catheter were introduced under the guidance of a 3D-EAM system. Ablation was performed at the anatomical PV ostium as defined by fast activation mapping (FAM), with ablation index (AI) targets of ≥400 at the posterior wall and ≥550 at the anterior wall, maintaining an interlesion spacing of ≤6 mm. Radiofrequency (RF) energy was delivered at 35 W for the posterior wall and 50 W for the anterior wall. All procedures utilized a Thermocool or Thermocool Surround Flow SF^®^ ablation catheter (Biosense Webster) with a saline irrigation flow rate of 17 mL/min. In repeat catheter ablation cases only reconnected PV (confirmed by EAM) were reisolated, until bidirectional block was achieved.

#### Anterior MIL with RFA

Anterior MIL ablation was performed using RF energy extending from the mitral annulus to the RSPV. The procedure was anatomically guided by fluoroscopy in the left anterior oblique view, along with a 3D-EAM system. Lesions were delivered until bidirectional block across the MI was achieved. MI block was confirmed by 3D-EAM and/or pacing from the left atrial appendage (LAA) using the ablation catheter, which resulted in a proximal-to-distal activation sequence on a decapolar catheter positioned in the coronary sinus.

#### Postprocedural care

For femoral hemostasis after the ablation, a figure of eight suture was applied followed by a compression dressing and 4 h of bed rest. Pericardial effusion was assessed by transthoracic echocardiography within one hour. After the intervention all patients received oral anticoagulation on the same day for a minimum of 8 weeks according to their CHA2DS2-VA score.

### Follow-up

The primary outcome was defined as freedom from AF or atrial tachycardia (AT) at 12 months following the index PVI, after a 60-day blanking period. Any documented AF, typical atrial flutter, atypical atrial flutter or AT episode lasting longer than 30 s was considered a recurrence. Secondary outcome encompassed procedural parameters such as total procedure time (measured from groin puncture to sheath removal), LA dwell time, and fluoroscopy time. Safety endpoints included phrenic nerve palsy, pericardial tamponade, transient ischaemic attack/stroke, and vascular access complications requiring surgical intervention. Follow-up was performed by the referral cardiologist and included a 24-hour Holter ECG after 3 and 6 months and a 7-day Holter ECG after 12 months. When the recurrence occurred during the blanking period and persisted, the date was set to 60 days after the initial intervention. If the patient underwent a ReDo-PVI, the number of reconnected PV and reconnected MIL were assessed.

### Statistical analysis

Continuous variables were reported as median and interquartile range (IQR), whereas categorical variables were presented as numbers and percentages. For the comparison of continuous variables, the Wilcoxon rank sum test was used and for categorical variables the Pearson’s Chi-squared test or Fisher’s exact test, as appropriate. To assess the probability of the arrhythmia-free survival the Kaplan Meier method was used combined with a log rank test. A *P*-value of 0.05 or less was considered as statistically significant. Statistical analyses were performed using R version 4.4.2 (The R Foundation for Statistical Computing, Vienna, Austria) and Rstudio 2024.12.1 + 563 (Posit Software PBC, Boston, MA, USA).

## Results

### Baseline characteristics

A total of 129 patients were included with a median age of 70 [64–76] years and 40% (52) of them were female. The median CHA2DS2-VA score was 2 [1–3]. A total of 61 patients (47%) received PFA, and 68 patients (53%) received RFA. 36% had paroxysmal AF and 64% persistent AF. Median left ventricular ejection fraction (LVEF) was 52 [44–59] % and median LA diameter was 43 [40–48] mm. There were no significant differences between the two groups except for the CHA2DS2-VA score (3 vs. 2 for PFA vs. RFA groups, respectively, *P* = 0.041) and the presence of diabetes mellitus (13% vs. 1% for PFA vs. RFA groups, respectively, *P* = 0.013). Baseline characteristics are summarized in *Table 1*.

**Table 1:** Patient characteristics at baseline

Baseline characteristics	Overall	PFA	RFA	*P*-value
	*n* = 129	*n* = 61	*n* = 68	
Age, years	70 [64–76]	71 [65–76]	69 [63–74]	0.063
Sex (female)	52 (40%)	25 (41%)	27 (40%)	0.883
BMI, kg/m^2^	27 [23–30]	27 [23–29]	26 [23–30]	0.510
LVEF, %	52 [44–59]	52 [43–58]	53 [44–59]	0.601
LA diameter, mm	43 [40–48]	44 [40–49]	42 [39–46]	0.108
Type of AF				0.408
Paroxysmal	46 (36%)	24 (39%)	22 (32%)	
Persistent	83 (64%)	37 (61%)	46 (68%)	
CHA2DS2-VA score	2 [1–3]	3 [1–3]	2 [1–3]	**0**.**041**
EHRA score				0.179
1	12 (13%)	8 (16%)	4 (10%)	
2a	18 (20%)	10 (20%)	8 (20%)	
2b	34 (38%)	21 (42%)	13 (33%)	
3	21 (23%)	7 (14%)	14 (35%)	
4	5 (6%)	4 (8%)	1 (2%)	
Hypertension	90 (70%)	44 (72%)	46 (67%)	0.580
Diabetes	9 (7%)	8 (13%)	1 (1%)	**0**.**013**
Hypercholesterolemia	43 (33%)	16 (26%)	27 (40%)	0.105
Coronary artery disease	16 (12%)	9 (15%)	7 (10%)	0.443
History of myocardial infarction	9 (7%)	5 (8%)	4 (6%)	0.735
Heart failure	21 (16%)	10 (16%)	11 (16%)	0.973
Smoking history	57 (44%)	28 (46%)	29 (43%)	0.710

Values are presented as median [interquartile range] or *n* (%).

AF, atrial fibrillation; LA, left atrial; LVEF, left ventricular ejection fraction; PFA, pulsed-field ablation; RFA, radiofrequency ablation.

### Procedural characteristics

At the beginning of the procedure, 36 patients (28%) were in AF, 47 (36%) in atypical atrial flutter or left AT and 39 (30%) in sinus rhythm. Of the 47 patients with atypical atrial flutter, 26 patients (53%) presented specifically with perimitral atrial flutter. There were no significant differences regarding the initial rhythm between the groups (*P* = 0.914). 67% and 84% of patients underwent repeat catheter ablation in the PFA and RFA group, respectively. Among them, 76% in the PFA group and 72% in the RFA group had durably isolated PVs on the preprocedural 3D-EAM (*P* = 0.127).

The total procedure duration for the PFA group was 71 [60–94] min and for the RFA group 108 [87–153] min, *P* < 0.001. LA dwell and fluoroscopy time in the PFA group were 53 [43–71] min and 9 [7–13] min vs. in the RFA group 80 [69–125] min and 8 [5–13] min, respectively, *P* < 0.001 and *P* = 0.037. Procedural duration remained significantly shorter for PFA compared with RFA when restricted to patients with durable PV isolation or a maximum of one reconnected PV and excluding other additional lesions than anterior MIL. Overall, 46 [29–60] applications (115 s) of PFA were applied in the PFA group and median RFA duration was 1073 [759–1770] seconds in the RFA group. To establish an anterior MIL, a median of 14 [10–23] PFA applications (35 s) were applied in the PFA group and a median RFA duration of 473 [253–733] s was applied in the RFA group, *P* < 0.001. Acute anterior MIL block was successful in all PFA cases and in 90% of the RFA cases. Further additional lesions with PWI being the most common were performed in 77 and 59% of the patients in the PFA and RFA group, respectively, *P* = 0.027 (*Table 2*). 10% in the PFA group and 24% in the RFA group underwent Cavotricuspid isthmus ablation (CTI), *P* = 0.039. Detailed procedural characteristics for patients undergoing repeat or first catheter ablation can be found in Supplementary material online, tables  *S1* and *S2*.

**Table 2:** Procedural characteristics

Procedural characteristics	Overall	PFA	RFA	*P*-value
	*n* = 129	*n* = 61	*n* = 68	
Total procedure duration, min	87 [65–114]	71 [60–94]	108 [87–153]	**<0**.**001**
LA Dwell time, min	68 [47–88]	53 [43–71]	80 [69–125]	**<0**.**001**
Ablation time, min	30 [17–52]	27 [16–35]	50 [23–85]	**<0**.**001**
Fluoroscopy time, min	9 [6–13]	9 [7–13]	8 [5–13]	**0**.**037**
Fluoroscopy dose, Gycm^2^	660 [350–1784]	473 [250–1200]	733 [415–2300]	**0**.**015**
Type of PVI				**0**.**021**
First	31 (24%)	20 (33%)	11 (16%)	
First ReDo	64 (49%)	30 (49%)	34 (50%)	
Second ReDo	28 (22%)	8 (13%)	20 (30%)	
Third ReDo	4 (3%)	3 (5%)	1 (1%)	
Fourth ReDo	2 (2%)	0 (0%)	2 (3%)	
Rhythm before ablation				0.914
AF	36 (28%)	19 (31%)	17 (25%)	
Typical AFlu	6 (5%)	3 (5%)	3 (4%)	
Atypical AFlu or AT	47 (36%)	22 (36%)	25 (37%)	
SR	39 (30%)	17 (28%)	22 (33%)	
Atrial Pacing	1 (1%)	0 (0%)	1 (1%)	
Number of transseptal punctures	1 [1–2]	1 [1–1]	2 [1–2]	**<0**.**001**
Perimitral flutter	26 (20%)	9 (15%)	17 (25%)	0.148
Reconnected LSPV	10 (10%)	5 (12%)	5 (9%)	0.738
Reconnected LIPV	7 (7%)	4 (10%)	3 (5%)	0.447
Reconnected RSPV	11 (11%)	1 (2%)	10 (18%)	**0**.**023**
Reconnected RIPV	10 (10%)	2 (5%)	8 (14%)	0.186
Number of reconnected PV				0.127
0	72 (73%)	31 (76%)	41 (72%)	
≥ 1	26 (27%)	10 (24%)	16 (28%)	
PV applications (PFA), Duration, s (RFA)	24 [0–340]	6 [0–32]	245 [0–928]	**<0**.**001**
MIL applications (PFA), Duration, s (RFA)	48 [14–488]	14 [10–23]	473 [253–733]	**<0**.**001**
Total applications (PFA), Duration, s (RFA)	258 [48–1112]	46 [29–60]	1073 [759–1770]	**<0**.**001**
Acute success of MIL	122 (95%)	61 (100%)	61 (90%)	**0**.**014**
Posterior wall isolation or roof line	87 (67%)	47 (77%)	40 (59%)	**0**.**027**
Extra PVI foci	2 (2%)	1 (2%)	1 (1%)	>0.999
Superior vena cava isolation	2 (2%)	0 (0%)	2 (3%)	0.498
Cavotricuspid isthmus ablation	22 (17%)	6 (10%)	16 (24%)	**0**.**039**
Hs-cTnT prior to PVI, ng/L	13 [9–19]	13 [8–21]	14 [11–19]	0.712
Hs-cTnT 1 day after PVI, ng/L	817 [474–1222]	935 [625–1579]	612 [263–859]	**0**.**001**

Values are presented as median [interquartile range] or *n* (%).

AF, atrial fibrillation; AFlu, atrial flutter; AT, left atrial tachycardia; Hs-cTnT, high-sensitive cardiac troponin T; LA, left atrial; MIL, mitral isthmus line; PFA, pulsed-field ablation; PV, pulmonary vein; PVI, pulmonary vein isolation; RFA, radiofrequency ablation; SR, sinus rhythm.

A total of 3 complications (3%) were observed. One stroke in the PFA group and two pericardial tamponades require drainage in the RFA group. The patient experiencing a stroke was admitted two days after the intervention with a left sided brachiocrural hemisyndrome [National Institutes of Health Stroke Scale (NIHSS) 14 points] and underwent interventional thrombectomy of the terminal right carotid artery and medial cerebral artery. Upon discharge to the rehabilitation a mild motoric brachiocrural hemisyndrome (NIHSS 2) persisted. No case of transient coronary spasm was observed.

### Arrhythmia-free survival

**Figure 1:**
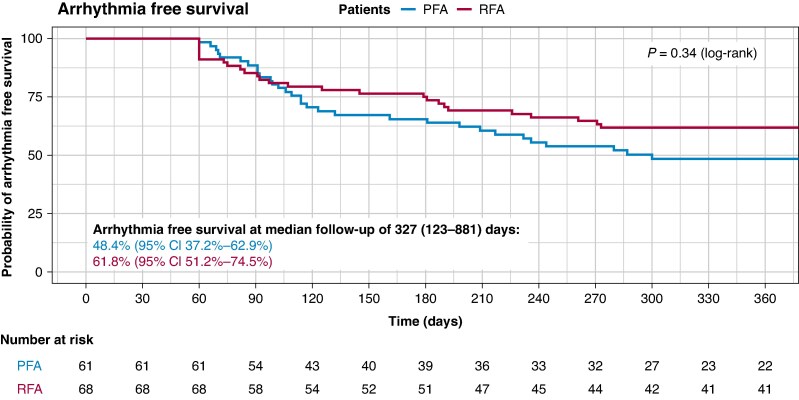
Kaplan Meier curve comparing the arrhythmia-free survival during one year in PFA and RFA. PFA, pulsed-field ablation; RFA, radiofrequency ablation.

The median follow-up duration was 327 days. At the time of the median follow-up and at 12 months, the arrhythmia-free survival rate was 48.4% for the PFA and 61.8% for the RFA group, *p*_log-rank_ = 0. 34 (*Figure 1*). 71 (55%) had recurrent atrial arrythmia. Among these, atypical atrial flutter or left AT were observed in 57% of the patients and AF in 42% of the patients with no difference between the two groups (*P* = 0.702). 13 patients (42%) undergoing a first catheter ablation had recurrent arrhythmia with AF in 54% and atypical atrial flutter or left AT in 36% of the cases (see Supplementary material online, *Table S4*). Among patients undergoing repeat catheter ablation (*n* = 98), arrythmia-free survival did not differ (41.0% vs. 63.2%, *P* = 0.10, Supplementary material online, *Figures S1* and *S2*). Among patients presenting with atypical atrial flutter or left AT at the beginning of the procedure, arrhythmia-free survival did not differ between the ablation modalities (see Supplementary material online, *Figures S3* and *S4*). 25 (53%) of patients presenting with atypical atrial flutter or left AT at the beginning of the procedure had recurrent arrhythmia. 72% of these patients had atypical atrial flutter or left AT as recurrent arrhythmia and 28% developed AF (see Supplementary material online, *Table S5*).

### Repeat catheter ablation after recurrence

**Figure 2:**
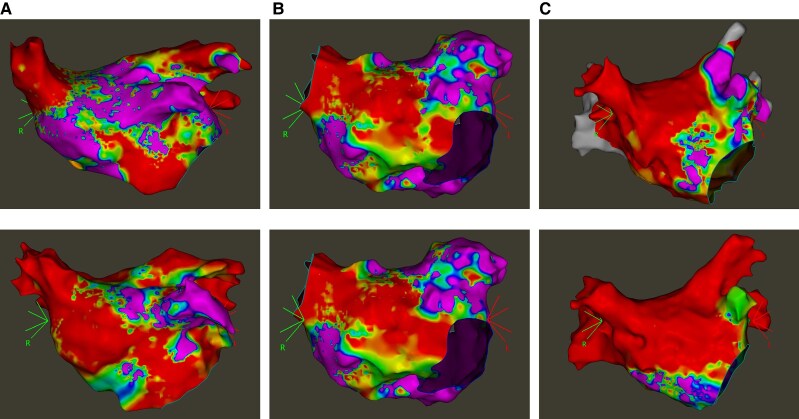
Examples of reconnected anterior mitral isthmus lines before and after ablation. *A*) Gap at the RSPV (RFA), *B*) gap at the anterior wall (PFA) and *C*) gap at the mitral annulus (PFA). PFA, pulsed-field ablation; RFA, radiofrequency ablation.

A total of 34 (48%) patients with arrhythmia recurrence underwent a redo procedure (12 patients in the PFA group and 22 in the RFA group). No reconnected PVs were found in 92% (11 patients) of the PFA group and 68% (15 patients) of the RFA group (*P* = 0.210). An incomplete anterior MIL was observed in 18 patients (53%). 8 patients (67%) in the PFA group vs. 10 patients (45%), in the RFA group (*P* = 0.236). The most frequent reconnected site was at the connection to the RSPV (33%). Further reconnection sites included the mitral annulus (20%) and anterior wall (27%) with no significant differences between the PFA group and the RFA group (*P* = 0.748) (*Table 3*). Examples of reconnected anterior MIL are provided in *Figure 2*. Among 3 out of 8 patients (38%) with recurrent atypical atrial flutter undergoing repeat catheter ablation, the arrhythmia mechanism was consistent with that observed at the index procedure.

**Table 3:** Efficacy and safety

Efficacy and Safety	Overall	PFA	RFA	*P*-value
	*n* = 129	*n* = 61	*n* = 68	
Recurrence	71 (55%)	31 (51%)	40 (59%)	0.362
Recurrence of any atrial arrhythmia				0.702
Atypical AFlu or AT	40 (57%)	17 (55%)	23 (57%)	
AF	30 (42%)	13 (42%)	17 (43%)	
Typical AFlu	1 (1%)	1 (3%)	0 (0%)	
Antiarrhythmic medication after PVI				0.135
After blanking period	1 (1%)	0 (0%)	1 (1%)	
During and after blanking period	20 (15%)	6 (10%)	14 (21%)	
During blanking period	22 (17%)	9 (15%)	13 (19%)	
None	86 (67%)	46 (75%)	40 (59%)	
Patients undergoing redo procedure	34 (26%)	12 (20%)	22 (32%)	0.103
Indication for ReDo				>0.999
Atypical AFlu or AT	21 (62%)	7 (59%)	14 (64%)	
AF	10 (29%)	4 (33%)	6 (27%)	
AF & atypical AFlu or AT	3 (9%)	1 (8%)	2 (9%)	
Number of reconnected PVs				0.210
0	26 (76%)	11 (92%)	15 (68%)	
≥ 1	8 (24%)	1 (8%)	7 (32%)	
Reconnected RSPV	3 (9%)	0 (0%)	3 (14%)	0.537
Reconnected LSPV	3 (9%)	0 (0%)	3 (14%)	0.537
Reconnected RIPV	5 (15%)	1 (8%)	4 (18%)	0.635
Reconnected LIPV	3 (9%)	0 (0%)	3 (14%)	0.537
Reconnected anterior MIL	18 (53%)	8 (67%)	10 (45%)	0.236
Site of Reconnection				0.748
RSPV	5 (33%)	2 (25%)	3 (43%)	
Annulus	3 (20%)	1 (13%)	2 (29%)	
Annulus & RSPV	3 (20%)	2 (25%)	1 (14%)	
Anterior wall	4 (27%)	3 (37%)	1 (14%)	
Complications				0.354
Pericardial tamponade	2 (2%)	0 (0%)	2 (3%)	
Stroke	1 (1%)	1 (2%)	0 (0%)	
None	126 (97%)	60 (98%)	66 (97%)	

Values are presented as median [interquartile range] or *n* (%).

AF, atrial fibrillation; AFlu, atrial flutter; AT, left atrial tachycardia; MIL, mitral isthmus line; PFA, pulsed-field ablation; PV, pulmonary vein; PVI, pulmonary vein isolation; RFA, radiofrequency ablation.

## Discussion

In this prospective, single-centre study, we compared PFA and RFA for PVI combined with anterior MIL ablation. Our results demonstrate that PFA significantly reduced procedural duration, and LA dwell time compared to RFA, while achieving comparable mid-term arrhythmia-free survival. However, the recurrence rate, particularly of AT and atypical flutter, remained high in both groups, underscoring the need for improved lesion durability along the MIL.

PFA was associated with a significantly shorter procedure time (71 vs. 108 min) and LA dwell time (53 vs. 80 min), which aligns with the efficiency advantages seen in the *MANIFEST-REDO* registry^17^ and other studies.^4,18–22^ These differences reflect the rapid lesion delivery and minimal catheter repositioning required in PFA. Additionally, anterior MIL creation using PFA required substantially fewer applications [median 14 (corresponding to 35 s) vs. 473 s RFA duration], further confirming its procedural advantages. Absolute time differences should be interpreted with cautions acknowledging anatomical and procedural heterogeneity inherent to redo cases.

The complication rate was low in both groups. These findings are in line with safety profiles from recent multicentre PFA studies, including *MANIFEST-17k*.^23^ Notably, data from the NCDR AFib Ablation Registry^24^ indicate that up to 4.5% of patients with persistent AF undergoing PVI plus additional lesions experienced minor or major complications. In contrast to a prior study using PFA for MIL,^13^ where two patients (4.4%) presented with reversible and non-fatal coronary spasm, we observed no such event.

Despite procedural efficiency, arrhythmia recurrence rates remained high (PFA 51%, RFA 59%), with AT or atypical atrial flutter accounting for 57% of recurrences. This aligns with *CAPLA*^5^ and *VENUS*,^6^ which highlight the limited incremental benefit of linear substrate modification over PVI in persistent AF. As emphasized in a recent EHRA consensus statement,^25^ macro-reeantrant ATs are common after extensive LA ablation and are often attributable to gaps in previously created ablation lines.

Anterior MIL reconnection was observed in 53% of patients undergoing redo ablation, with no significant difference between PFA and RFA groups. This finding challenges recent data from Davong et al.^13^ who reported high rates of acute MIL block with PFA, suggesting that while acute success may be achievable, long-term lesion durability remains insufficient. In a recent pilot study^26^ evaluating focal PFA application in 54 patients undergoing redo procedures, the anterior mitral line showed the highest rate of conduction gaps among all linear lesions during follow-up redo ablation, with 6/7 anterior lines demonstrating reconduction, underscoring the limited durability of lesions in this area despite initial procedural success. Reconnection of the MIL in this study often occurred at the RSPV or mitral annulus—locations where PFA catheter contact is anatomically suboptimal—especially with pentaspline systems not optimized for linear ablation. Cubberley et al.^14^ demonstrated improved MIL block durability when RFA was combined with ethanol infusion of the VOM, indicating that multimodal ablation strategies may be required to overcome anatomic limitations. Similarly, in the stepwise approach reported by Li et al.^27^ additional epicardial ablation within the CS and VOM resulted in higher acute success and arrhythmia-free survival of 82% during follow-up. New focal ablation catheters capable of delivering both PFA and RFA may further optimize lesion formation by combining the advantages of both energy sources.^28^ This integrated approach might enhance the durability of linear lesions, particularly in challenging regions such as the anterior MI.

Taken together, these findings highlight the limitations of empiric anterior MIL ablation. The heterogeneity of indications, the lack of proven incremental benefit beyond PVI, and the high rate of reconnection observed at redo procedures all argue for a patient-tailored ablation strategy and randomized controlled trials to better define the role of anterior MIL ablation.

Similar to *CAPLA*,^5^ this study questions the benefit of empiric linear ablation in patients without proven macro-reentrant circuits. Furthermore, in our cohort, a large proportion of patients (36%) presented with atypical flutters or AT pre-ablation, potentially reflecting prior ablation-related scarring and the limitations of conventional lesion sets. Given the modest arrhythmia-free survival despite efficient PFA, future research should focus on tailored substrate modification guided by high-density mapping or fibrosis imaging, rather than routine use of linear ablation, also in patients undergoing repeat catheter ablation.

This study is limited by its non-randomized, single-centre design and differences in temporal inclusion, as PFA was introduced only in 2023. This discrepancy may introduce procedural era bias. Additionally, not all patients underwent continuous rhythm monitoring, which may underestimate true recurrence rates. Anterior MIL was performed using a pentaspline catheter not optimized for linear ablation and thus cannot be translated to other PFA platforms. Further studies assessing focal tip PFA catheters as well as integrated mapping function are warranted. Similarly, intracardiac echocardiography (ICE) was not routinely used in all cases to confirm tissue contact. We evaluated in this study an anterior MIL extending from the mitral annulus to the RSPV. These findings are specific to this anatomical configuration and should not be extrapolated to other MIL strategies, such as those extending from the LIPV to the mitral annulus or from the LAA to the mitral annulus. Furthermore, a substantial proportion of patients in both groups underwent additional PWI. This concomitant lesion set may have impacted arrhythmia outcomes. Lastly, no systematic waiting time was applied in both groups, which may overestimate acute success, especially for PFA.^13^

In conclusion, anterior MIL ablation using PFA is feasible, safe and significantly more time-efficient than RFA. However, long-term efficacy remains limited, primarily due to recurrent macro-reentrant arrhythmias and frequent MIL reconnection. These findings emphasize the need for improved catheter designs and refined, patient-tailored ablation strategies. Randomized controlled trials are warranted to evaluate the utility and durability of linear lesions created with PFA in persistent AF and patients undergoing repeat CA.

## Supplementary Material

euaf265_Supplementary_Data

## Data Availability

The data underlying this article will be shared on reasonable request to the corresponding author.
