# Supplementary material for: Anterior mitral isthmus line using pulsed-field ablation with the pentaspline catheter or radiofrequency ablation: procedural characteristics, safety, and mid-term outcomes
Source: Europace. 2025 Oct 23;27(11):euaf265. doi: 10.1093/europace/euaf265 (PMC12585187; doi:10.1093/europace/euaf265)
Supplement: euaf265_Supplementary_Data [file euaf265_supplementary_data.docx]

**Supplementary Table S1**

| **Procedural characteristics ReDo** | **Overall** N = 98 | **PFA** N = 41 | **RFA** N = 57 | **p-value** |
| --- | --- | --- | --- | --- |
| **Total procedure duration, min** | 88 [66 - 118] | 71 [60 - 90] | 114 [87 - 153] | **<0.001** |
| **LA Dwell time, min** | 69 [47 - 93] | 53 [43 - 70] | 79 [69 - 125] | **<0.001** |
| **Ablation time, min** | 29 [17 - 53] | 21 [13 - 32] | 50 [23 - 85] | **<0.001** |
| **Fluoroscopy time, min** | 8 [5 - 12] | 9 [7 - 13] | 7 [5 - 12] | 0.088 |
| **Fluoroscopy dose, Gycm^2^** | 652 [362 - 1,586] | 525 [324 - 1,178] | 730 [415 - 2,163] | 0.127 |
| **Type of PVI** |  |  |  | 0.101 |
| First | 0 (0%) | 0 (0%) | 0 (0%) |  |
| 1st ReDo | 64 (65%) | 30 (73%) | 34 (59%) |  |
| 2nd ReDo | 28 (29%) | 8 (20%) | 20 (35%) |  |
| 3rd ReDo | 4 (4%) | 3 (7%) | 1 (2%) |  |
| 4th ReDo | 2 (2%) | 0 (0%) | 2 (4%) |  |
| **Rhythm before ablation** |  |  |  | 0.952 |
| AF | 27 (27%) | 13 (32%) | 14 (24%) |  |
| Typical AFlu | 3 (3%) | 1 (2%) | 2 (4%) |  |
| Atypical AFlu or AT | 38 (39%) | 15 (37%) | 23 (40%) |  |
| SR | 29 (30%) | 12 (29%) | 17 (30%) |  |
| Atrial Pacing | 1 (1%) | 0 (0%) | 1 (2%) |  |
| **Number of transseptal punctures** | 1 [1 - 2] | 1 [1 - 1] | 2 [1 - 2] | **<0.001** |
| **Perimitral flutter** | 20 (20%) | 5 (12%) | 15 (26%) | 0.087 |
| **Reconnected LSPV** | 10 (10%) | 5 (12%) | 5 (9%) | 0.738 |
| **Reconnected LIPV** | 7 (7%) | 4 (10%) | 3 (5%) | 0.447 |
| **Reconnected RSPV** | 11 (11%) | 1 (2%) | 10 (18%) | **0.023** |
| **Reconnected RIPV** | 10 (10%) | 2 (5%) | 8 (14%) | 0.186 |
| **Number of reconnected PV** |  |  |  | 0.127 |
| 0 | 72 (73%) | 31 (76%) | 41 (72%) |  |
| ≥1 | 26 (27%) | 10 (24%) | 16 (28%) |  |
| **PV applications (PFA), Duration, s (RFA)** | 4 [0 - 222] | 0 [0 - 6] | 180 [0 - 641] | **<0.001** |
| **MIL applications (PFA), Duration, s (RFA)** | 213 [20 - 553] | 18 [10 - 24] | 500 [266 - 761] | **<0.001** |
| **Total applications (PFA), Duration, s (RFA)** | 606 [46 - 1,081] | 36 [28 - 50] | 1,017 [749 - 1,608] | **<0.001** |
| **Acute success of MIL** | 92 (94%) | 41 (100%) | 51 (89%) | **0.039** |
| **Posterior wall isolation or roof line** | 68 (69%) | 32 (78%) | 36 (63%) | 0.115 |
| **Extra PVI foci** | 2 (2%) | 1 (2%) | 1 (2%) | >0.999 |
| **Superior vena cava isolation** | 1 (1%) | 0 (0%) | 1 (2%) | >0.999 |
| **Cavotricuspid isthmus ablation** | 15 (15%) | 3 (7%) | 12 (21%) | 0.062 |
| **Hs-cTnT prior to PVI, ng/L** | 13 [9 - 18] | 11 [7 - 19] | 13 [11 - 18] | 0.220 |
| **Hs-cTnT 1 day after PVI, ng/L** | 646 [379 - 1,011] | 835 [467 - 1,188] | 565 [256 - 768] | **0.021** |

**Supplementary Table S1:** Procedural characteristics ReDo cases. Values are presented as median [interquartile range] or n (%).
AF = atrial fibrillation; AFlu = atrial flutter; AT = left atrial tachycardia; Hs-cTnT = high-sensitive cardiac troponin T; LA = left atrial; MIL = mitral isthmus line; PFA = pulsed-field ablation; PV = pulmonary vein; PVI = pulmonary vein isolation; RFA = radiofrequency ablation; SR = sinus rhythm.

**Supplementary Table S2**

| **Procedural characteristics First** | **Overall**  N = 31 | **PFA**  N = 20 | **RFA**  N = 11 | **p-value** |
| --- | --- | --- | --- | --- |
| **Total procedure duration, min** | 73 [63 - 101] | 72 [63 - 97] | 99 [77 - 150] | 0.295 |
| **LA Dwell time, min** | 54 [45 - 79] | 53 [45 - 75] | 84 [61 - 130] | 0.175 |
| **Ablation time, min** | 36 [27 - 45] | 33 [27 - 44] | 52 [33 - 110] | 0.245 |
| **Fluoroscopy time, min** | 12 [7 - 16] | 12 [8 - 15] | 12 [5 - 23] | 0.820 |
| **Fluoroscopy dose, Gycm^2^** | 660 [238 - 1,973] | 397 [226 - 1,457] | 2,488 [679 - 5,723] | **0.040** |
| **Type of PVI** |  |  |  | >0.999 |
| First | 31 (100%) | 20 (100%) | 11 (100%) |  |
| **Rhythm before ablation** |  |  |  | 0.760 |
| AF | 9 (29%) | 6 (30%) | 3 (27%) |  |
| Typical AFlu | 3 (10%) | 2 (10%) | 1 (9%) |  |
| Atypical AFlu or AT | 9 (29%) | 7 (35%) | 2 (18%) |  |
| SR | 10 (32%) | 5 (25%) | 5 (46%) |  |
| Atrial Pacing | 0 (0%) | 0 (0%) | 0 (0%) |  |
| **Number of transseptal punctures** | 1 [1 - 1] | 1 [1 - 1] | 2 [1 - 2] | **0.002** |
| **Perimitral flutter** | 6 (19%) | 4 (20%) | 2 (18%) | >0.999 |
| **PV applications (PFA), Duration, s (RFA)** | 48 [32 - 993] | 33 [28 - 45] | 1,334 [913 - 2,316] | **<0.001** |
| **MIL applications (PFA), Duration, s (RFA)** | 16 [11 - 171] | 12 [8 - 17] | 458 [160 - 710] | **<0.001** |
| **Total applications (PFA), Duration, s (RFA)** | 74 [55 - 1,394] | 63 [51 - 73] | 2,207 [1,379 - 2,926] | **<0.001** |
| **Acute success of MIL** | 30 (97%) | 20 (100%) | 10 (91%) | 0.355 |
| **Posterior wall isolation or roof line** | 19 (61%) | 15 (75%) | 4 (36%) | 0.056 |
| **Extra PVI foci** | 0 (0%) | 0 (0%) | 0 (0%) |  |
| **Superior vena cava isolation** | 1 (3%) | 0 (0%) | 1 (9%) | 0.355 |
| **Cavotricuspid isthmus ablation** | 7 (23%) | 3 (15%) | 4 (36%) | 0.210 |
| **Hs-cTnT prior to PVI, ng/L** | 15 [11 - 28] | 15 [11 - 33] | 17 [9 - 23] | 0.816 |
| **Hs-cTnT 1 day after PVI, ng/L** | 1,556 [989 - 1,977] | 1,651 [985 - 2,603] | 1,051 [989 - 1,159] | 0.307 |

**Supplementary Table S2:** Procedural characteristics First cases. Values are presented as median [interquartile range] or n (%).
AF = atrial fibrillation; AFlu = atrial flutter; AT = left atrial tachycardia; Hs-cTnT = high-sensitive cardiac troponin T; LA = left atrial; MIL = mitral isthmus line; PFA = pulsed-field ablation; PV = pulmonary vein; PVI = pulmonary vein isolation; RFA = radiofrequency ablation; SR = sinus rhythm.

| **Efficacy and Safety ReDo** | **Overall** N = 98 | **PFA** N = 41 | **RFA** N = 57 | **p-value** |
| --- | --- | --- | --- | --- |
| **Recurrence** | 58 (59%) | 24 (59%) | 34 (60%) | 0.912 |
| **Recurrence of any atrial arrhythmia** |  |  |  | >0.999 |
| Atypical AFlu or AT | 35 (60%) | 14 (58%) | 21 (62%) |  |
| AF | 23 (40%) | 10 (42%) | 13 (38%) |  |
| **Antiarrhythmic medication after PVI** |  |  |  | 0.254 |
| after blanking period | 1 (1%) | 0 (0%) | 1 (2%) |  |
| during and after blanking period | 16 (16%) | 4 (10%) | 12 (21%) |  |
| during blanking period | 19 (20%) | 7 (17%) | 12 (21%) |  |
| none | 62 (63%) | 30 (73%) | 32 (56%) |  |
| **Patients undergoing redo procedure** | 25 (26%) | 9 (22%) | 16 (28%) | 0.493 |
| **Indication for ReDo** |  |  |  | 0.803 |
| Atypical AFlu or AT | 19 (76%) | 6 (67%) | 13 (81%) |  |
| AF | 4 (16%) | 2 (22%) | 2 (13%) |  |
| AF & atypical AFlu or AT | 2 (8%) | 1 (11%) | 1 (6%) |  |
| **Number of reconnected PVs** |  |  |  | >0.999 |
| 0 | 21 (84%) | 8 (89%) | 13 (81%) |  |
| ≥1 | 4 (16%) | 1 (11%) | 3 (19%) |  |
| **Reconnected RSPV** | 1 (4%) | 0 (0%) | 1 (6%) | >0.999 |
| **Reconnected LSPV** | 1 (4%) | 0 (0%) | 1 (6%) | >0.999 |
| **Reconnected RIPV** | 2 (8%) | 1 (11%) | 1 (6%) | >0.999 |
| **Reconnected LIPV** | 0 (0%) | 0 (0%) | 0 (0%) | >0.999 |
| **Reconnected anterior MIL** | 15 (60%) | 7 (78%) | 8 (50%) | 0.229 |
| **Site of Reconnection** |  |  |  | 0.685 |
| RSPV | 5 (39%) | 2 (28.5%) | 3 (50%) |  |
| Annulus | 3 (23%) | 1 (14.5%) | 2 (33%) |  |
| Annulus & RSPV | 3 (23%) | 2 (28.5%) | 1 (17%) |  |
| Anterior wall | 2 (15%) | 2 (28.5%) | 0 (0%) |  |
| **Complications** |  |  |  | 0.316 |
| Pericardial tamponade | 2 (2%) | 0 (0%) | 2 (4%) |  |
| Stroke | 1 (1%) | 1 (2%) | 0 (0%) |  |
| none | 95 (97%) | 40 (98%) | 55 (96%) |  |

**Supplementary Table S3**

**Supplementary Table S3:** Efficacy and Safety ReDo cases. Values are presented as median [interquartile range] or n (%).
AF = atrial fibrillation; AFlu = atrial flutter; AT = left atrial tachycardia; MIL = mitral isthmus line; PFA = pulsed-field ablation; PV = pulmonary vein; PVI = pulmonary vein isolation; RFA = radiofrequency ablation.

**Supplementary Table S4**

| **Efficacy and Safety First** | **Overall** N = 31 | **PFA** N = 20 | **RFA** N = 11 | **p-value** |
| --- | --- | --- | --- | --- |
| **Recurrence** | 13 (42%) | 7 (35%) | 6 (55%) | 0.449 |
| **Recurrence of any atrial arrhythmia** |  |  |  | >0.999 |
| Atypical AFlu or AT | 5 (38%) | 3 (43%) | 2 (33%) |  |
| AF | 7 (54%) | 3 (43%) | 4 (67%) |  |
| Typical AFlu | 1 (8%) | 1 (14%) | 0 (0%) |  |
| **Antiarrhythmic medication after PVI** |  |  |  | 0.815 |
| during and after blanking period | 4 (13%) | 2 (10%) | 2 (18%) |  |
| during blanking period | 3 (10%) | 2 (10%) | 1 (9%) |  |
| none | 24 (77%) | 16 (80%) | 8 (73%) |  |
| **Patients undergoing redo procedure** | 9 (29%) | 3 (15%) | 6 (55%) | **0.038** |
| **Indication for ReDo** |  |  |  | >0.999 |
| Atypical AFlu or AT | 2 (22%) | 1 (33%) | 1 (17%) |  |
| AF | 6 (67%) | 2 (67%) | 4 (66%) |  |
| AF & atypical AFlu or AT | 1 (11%) | 0 (0%) | 1 (17%) |  |
| **Number of reconnected PVs** |  |  |  | 0.167 |
| 0 | 5 (56%) | 3 (100%) | 2 (33%) |  |
| ≥1 | 4 (44%) | 0 (0%) | 4 (67%) |  |
| **Reconnected RSPV** | 2 (22%) | 0 (0%) | 2 (33%) | 0.500 |
| **Reconnected LSPV** | 2 (22%) | 0 (0%) | 2 (33%) | 0.500 |
| **Reconnected RIPV** | 3 (33%) | 0 (0%) | 3 (50%) | 0.464 |
| **Reconnected LIPV** | 3 (33%) | 0 (0%) | 3 (50%) | 0.464 |
| **Reconnected anterior MIL** | 3 (33%) | 1 (33%) | 2 (33%) | >0.999 |
| **Site of Reconnection** |  |  |  | >0.999 |
| Anterior wall | 2 (100%) | 1 (100%) | 1 (100%) |  |
| **Complications** |  |  |  | >0.999 |
| none | 31 (100%) | 20 (100%) | 11 (100%) |  |

**Supplementary Table S4:** Efficacy and Safety First cases. Values are presented as median [interquartile range] or n (%).
AF = atrial fibrillation; AFlu = atrial flutter; AT = left atrial tachycardia; MIL = mitral isthmus line; PFA = pulsed-field ablation; PV = pulmonary vein; PVI = pulmonary vein isolation; RFA = radiofrequency ablation.

**Supplementary Table S5**

| **Efficacy and Safety AT** | **Overall** N = 47 | **PFA** N = 22 | **RFA** N = 25 | **p-value** |
| --- | --- | --- | --- | --- |
| **Recurrence** | 25 (53%) | 10 (45%) | 15 (60%) | 0.319 |
| **Recurrence of any atrial arrhythmia** |  |  |  | >0.999 |
| Atypical AFlu or AT | 18 (72%) | 7 (70%) | 11 (73%) |  |
| AF | 7 (28%) | 3 (30%) | 4 (27%) |  |
| **Antiarrhythmic medication after PVI** |  |  |  | 0.726 |
| during and after blanking period | 4 (9%) | 1 (4%) | 3 (12%) |  |
| during blanking period | 11 (23%) | 5 (23%) | 6 (24%) |  |
| none | 32 (68%) | 16 (73%) | 16 (64%) |  |
| **Patients undergoing redo procedure** | 10 (21%) | 3 (14%) | 7 (28%) | 0.297 |
| **Indication for ReDo** |  |  |  | >0.999 |
| Atypical AFlu or AT | 8 (80%) | 2 (67%) | 6 (86%) |  |
| AF | 2 (20%) | 1 (33%) | 1 (14%) |  |
| **Number of reconnected PVs** |  |  |  | >0.999 |
| 0 | 8 (80%) | 3 (100%) | 5 (71%) |  |
| ≥1 | 2 (20%) | 0 (0%) | 2 (29%) |  |
| **Reconnected RSPV** | 0 (0%) | 0 (0%) | 0 (0%) | >0.999 |
| **Reconnected LSPV** | 1 (10%) | 0 (0%) | 1 (14%) | >0.999 |
| **Reconnected RIPV** | 1 (10%) | 0 (0%) | 1 (14%) | >0.999 |
| **Reconnected LIPV** | 0 (0%) | 0 (0%) | 0 (0%) | >0.999 |
| **Reconnected anterior MIL** | 7 (70%) | 3 (100%) | 4 (57%) | 0.475 |
| **Site of Reconnection** |  |  |  | 0.600 |
| Annulus | 1 (20%) | 0 (0%) | 1 (50%) |  |
| Annulus & RSPV | 2 (40%) | 1 (33%) | 1 (50%) |  |
| Anterior wall | 2 (40%) | 2 (67%) | 0 (0%) |  |
| **Complications** |  |  |  | >0.999 |
| Pericardial tamponade | 1 (2%) | 0 (0%) | 1 (4%) |  |
| none | 46 (98%) | 22 (100%) | 24 (96%) |  |

**Supplementary Table S5:** Efficacy and Safety presenting in atypical AFlu or AT at the beginning of the intervention. Values are presented as median [interquartile range] or n (%).
AF = atrial fibrillation; AFlu = atrial flutter; AT = left atrial tachycardia; MIL = mitral isthmus line; PFA = pulsed-field ablation; PV = pulmonary vein; PVI = pulmonary vein isolation; RFA = radiofrequency ablation.

**Supplementary Figure S1
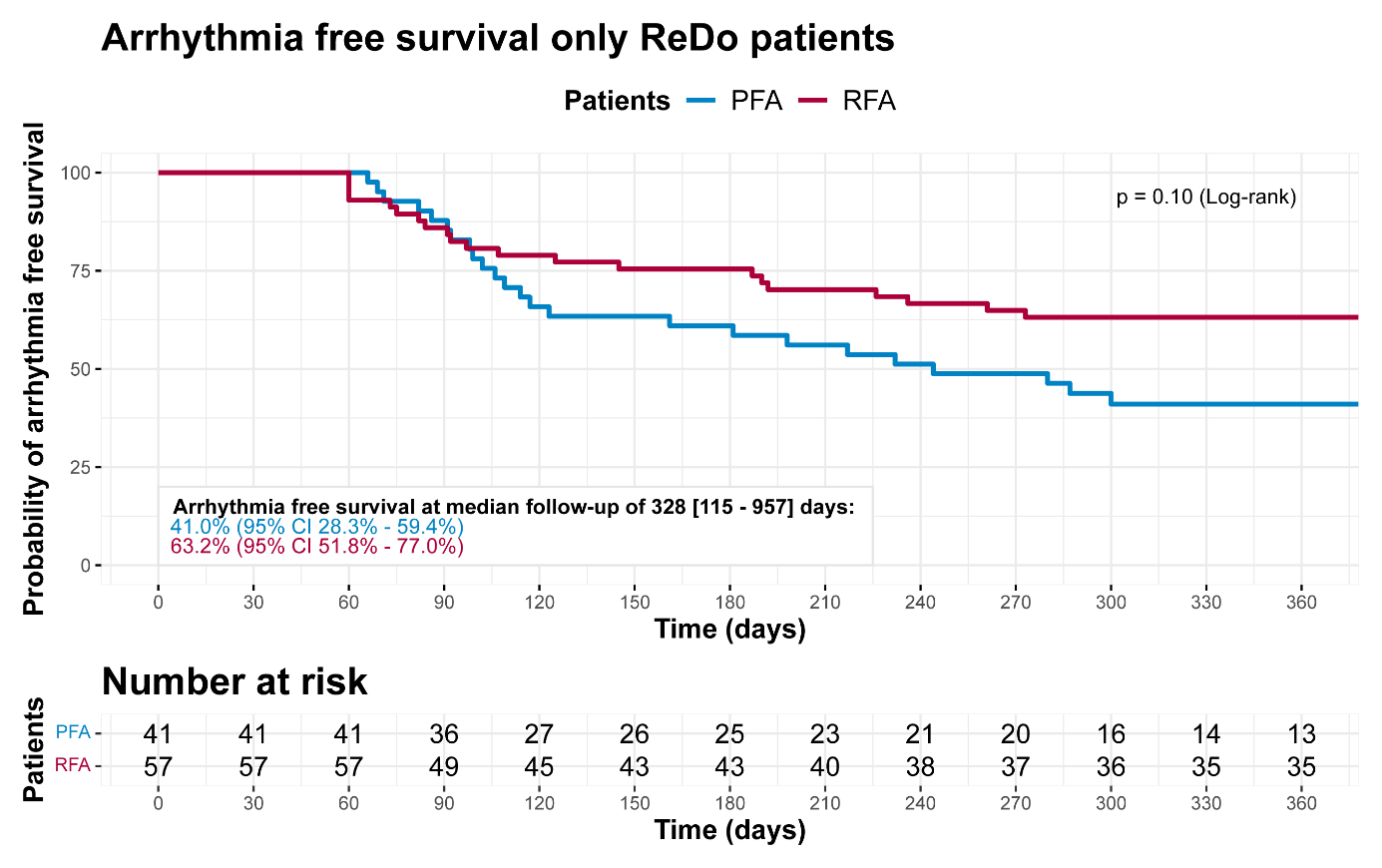
**

**Supplementary Figure S1:** Kaplan Meier curve comparing the arrhythmia free survival during one year in PFA and RFA in patients undergoing a ReDo intervention. PFA = pulsed-field ablation; RFA = radiofrequency ablation.

**Supplementary Figure S2**


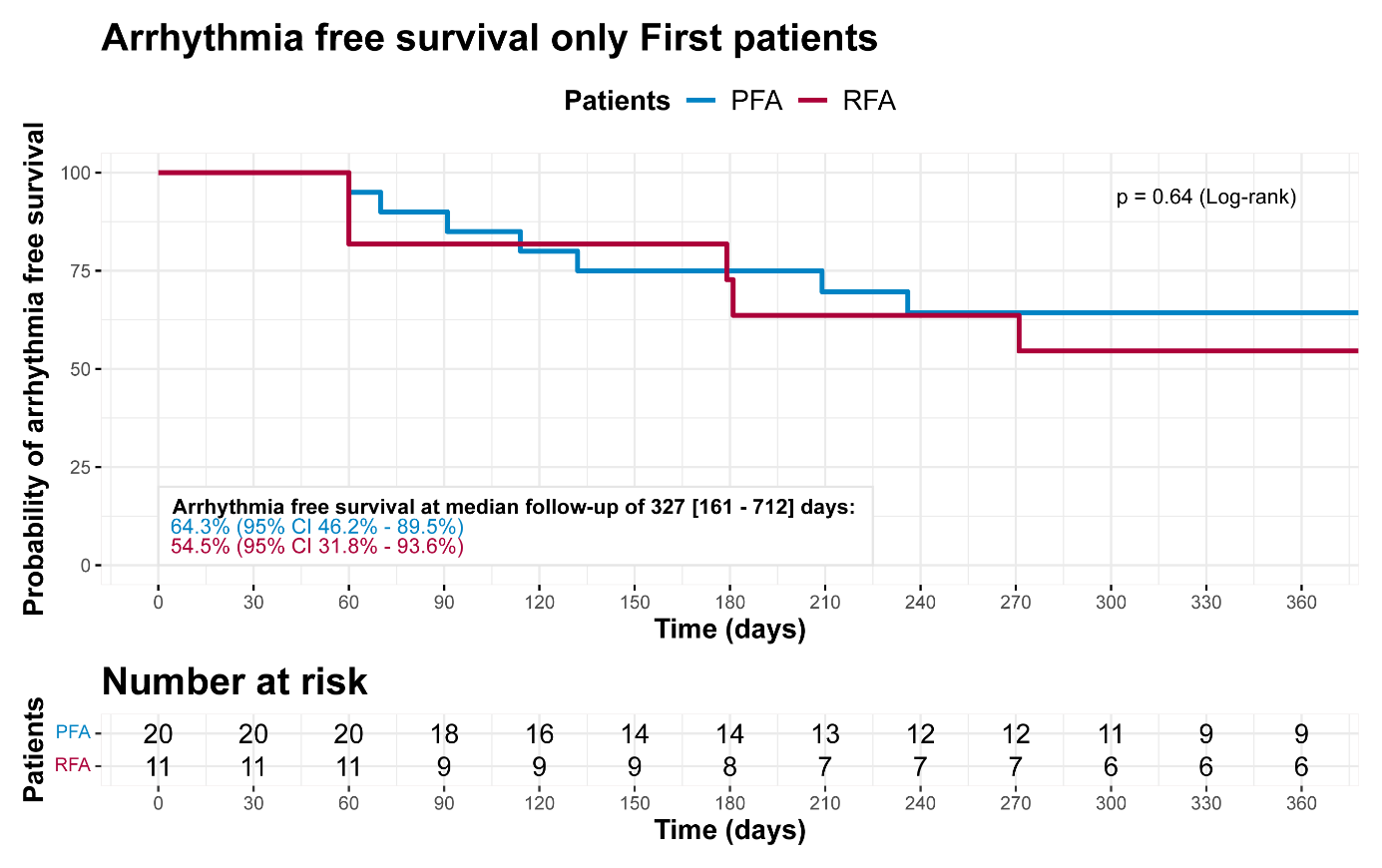


**Supplementary Figure S2:** Kaplan Meier curve comparing the arrhythmia free survival during one year in PFA and RFA in patients undergoing a First intervention. PFA = pulsed-field ablation; RFA = radiofrequency ablation.

**Supplementary Figure S3**

**
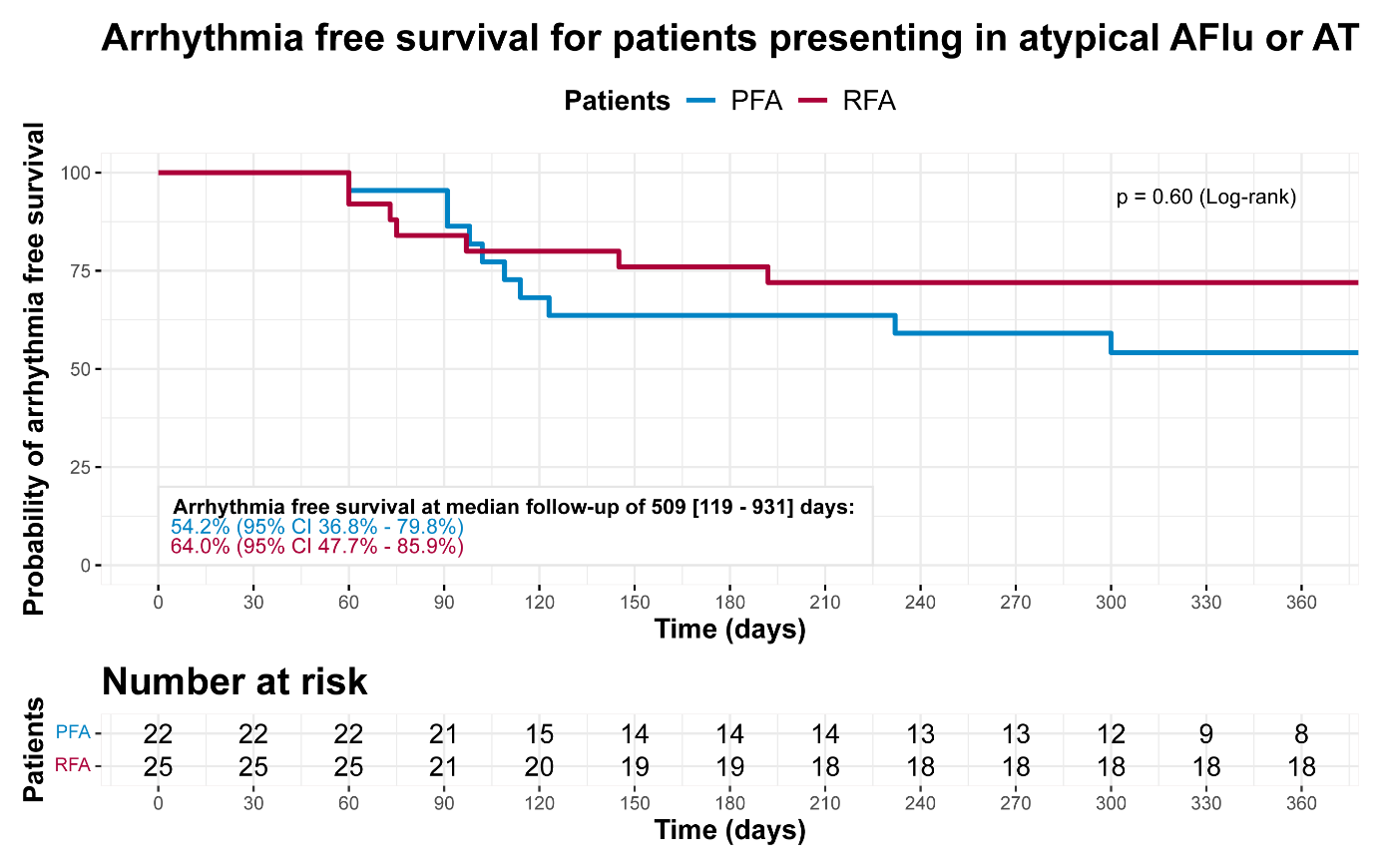
Supplementary Figure S3:** Kaplan Meier curve comparing the arrhythmia free survival during one year in PFA and RFA in patients presenting in atypical AFlu or AT at the beginning of the intervention. AFlu = atrial flutter; AT = left atrial tachycardia; PFA = pulsed-field ablation; RFA = radiofrequency ablation.

**Supplementary Figure S4
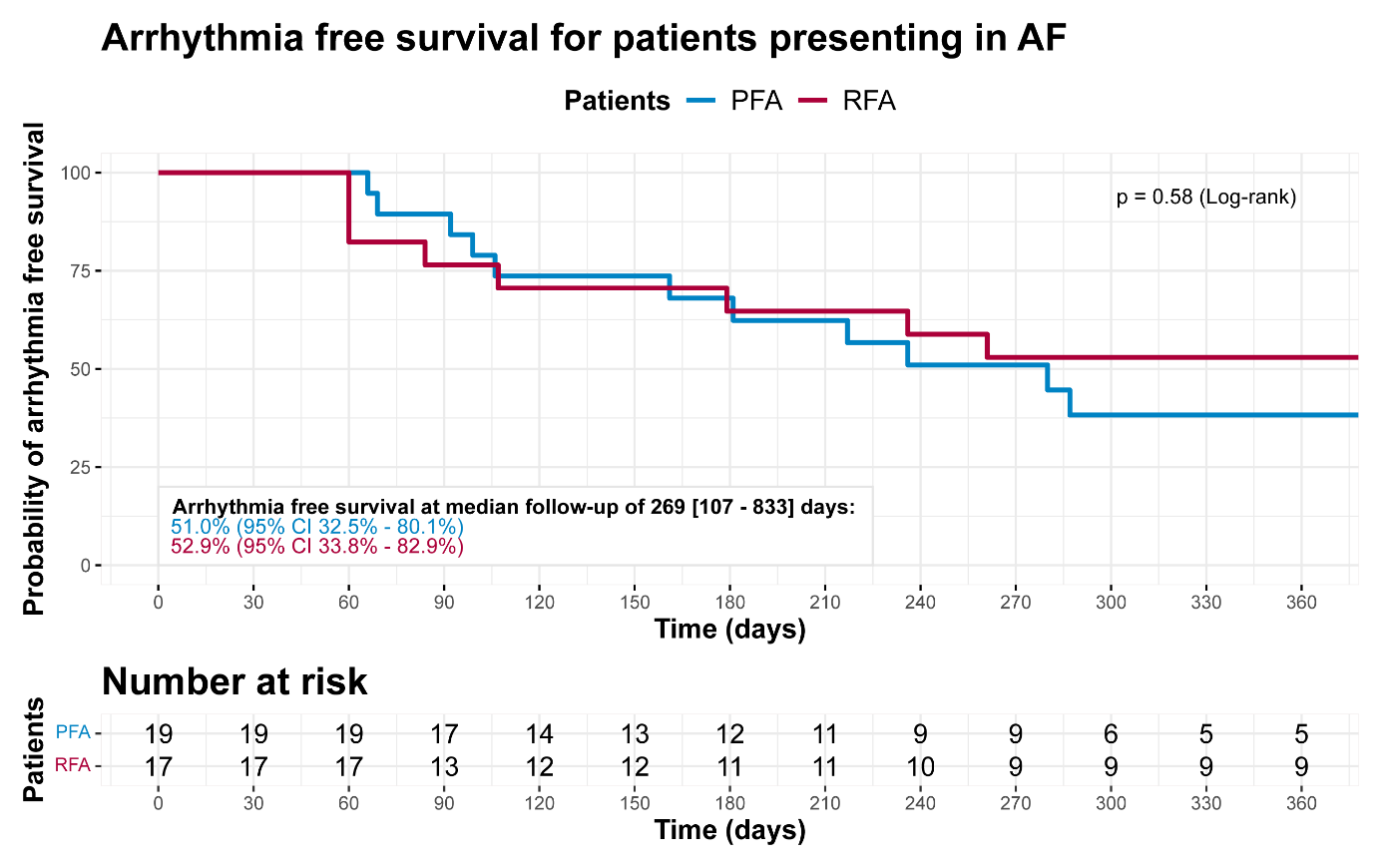
**

**Supplementary Figure S4:** Kaplan Meier curve comparing the arrhythmia free survival during one year in PFA and RFA in patients presenting in AF at the beginning of the intervention. AF = atrial fibrillation; PFA = pulsed-field ablation; RFA = radiofrequency ablation.
